# Co‐housing with Alzheimer's disease mice induces changes in gut microbiota and impairment of learning and memory in control mice

**DOI:** 10.1111/cns.14491

**Published:** 2023-10-03

**Authors:** Jun Li, Weiran Shan, Zhiyi Zuo

**Affiliations:** ^1^ Department of Anesthesiology University of Virginia Charlottesville Virginia USA

Alzheimer's disease (AD) is the most common form of dementia in the elderly.^1^ The clinical presentation of AD includes progressive impairment of cognition, attention, orientation, and logic. The pathological hallmarks in the brain of a patient with AD are extracellular amyloid plaques formed by accumulated β‐amyloid peptide (Aβ), an enzymatic product of amyloid precursor protein,^2^, ^3^ and intracellular neurofibrillary tangles consisting of hyperphosphorylated tau protein.^4^ Recently, it has been shown that patients with AD have gut microbiota that is different from control subjects.^5^, ^6^, ^7^ Animals with AD‐like brain changes have altered gut microbiota that increases inflammatory responses.^8^ Replacing gut microbiota of AD‐like mice with that of healthy mice attenuates AD‐like brain and behavior changes.^9^ These findings suggest that gut microbiota plays a role in AD‐like brain changes.

It has been reported that spousal caregivers of patients with dementia have an increased risk for dementia later in life.^10^ Although the mechanism for this phenomenon is not clear, increased stress due to caregiving to the patients and similar living environment are thought to contribute to this phenomenon.^10^, ^11^ It is possible that the patients with dementia and their spouses have some shared gut microbiota, which may then induce cascade events to ultimately lead to brain changes for dementia later in life. Thus, we hypothesize that living with subjects with dementia can lead to gut microbiota changes and impairment of learning and memory in control subjects. As the first step to test this hypothesis, we co‐housed control mice with mice carrying AD genes and examined their gut microbiota and permeability.

Six‐month‐old female 3xTg‐AD mice [B6;129‐Tg (APPSwe, tauP301L) 1Lfa *Psen1* ^*tm1Mpm*^/Mmjax, Jackson Laboratory] and the same age and gender control mice (B6129SF2/J, Jackson Laboratory) were randomly assigned to the following groups: (1) control group (only control mice in a cage), (2) AD group (only 3xTg‐AD mice in a cage), and (3) co‐housing group (control mice were co‐housed with 3xTg‐AD mice). For the co‐housing condition, 2 or 3 AD mice were co‐housed with 3 or 2 control mice, respectively, to have 5 mice in each cage for 8 months. Control group and AD group also had 5 mice per cage.

The learning and memory of the mice were tested by Barnes maze and fear conditioning as we described before.^12^, ^13^ Barnes maze tests spatial learning and memory. Fear conditioning examines hippocampus‐dependent (context‐related fear conditioning) and hippocampus‐independent (tone‐related fear conditioning) learning and memory.^14^ To determine the intestinal permeability assay, mice were fasted for 6 h before test, and gavaged with fluorescein isothiocyanate (FITC)‐dextran (4 KDa, catalog number: 46944‐500MG‐F, Sigma) at 600 mg/kg body weight. The blood was collected 2 h after gavage and the plasma was prepared for fluorescence measurement at excitation = 485 nm and emission = 530 nm. Feces were collected for 16S rDNA metagenomics study. Total DNA was extracted with DNeasy Powerlyzer Powersoil kit (#12855, Qiagen) and sent to LC Sciences for 16S rDNA sequencing. The primers (341F/805R) designed to target the V3 and V4 regions of 16S rDNA generated an amplicon about 465 bp in size. The amplified library was sequenced on a NovaSeq platform with 250 bp paired‐end reads mode (2 × 250 bp). The relative abundance of taxa within gut microbiota at genus level was compared between groups. The gut microbiota data analysis was performed by LC Sciences.

The results of training sessions of Barnes tests were analyzed by one‐way repeated measures ANOVA for data within one group or two‐way repeated measures ANOVA for the comparison between groups. One‐way ANOVA on rank followed by Tukey's multiple comparison test was used to analyze significance of the other data. Shapiro–Wilk test was used to examine the normal distribution of the data.

The age‐matched control mice took less time to identify the target box with more training sessions. The time for AD mice, co‐housed control mice and co‐housed AD mice to identify the target box on the fourth training day was shorter than that for those mice on the first day, while the time for control mice to identify the target box on the third and fourth training days was shorter than that on the first day (Figure 1). Having AD genes was a significant factor to increase the time to identify the target box during the training sessions [*F*(1,18) = 5.785, *p* = 0.027]. Similarly, co‐housing with AD mice was a significant factor to increase the time for control mice to identify the target box during the training sessions [*F*(1,8) = 27.753, *p* < 0.001] but co‐housing with control mice was not a factor to affect the time for AD mice to identify the target box during the training sessions [*F*(1,8) = 0.0099, *p* = 0.923]. In addition, AD mice, co‐housed control mice and co‐housed AD mice took longer times than control mice to identify the target box 1 and 8 days after the training sessions. AD mice and co‐housed control mice had less freezing behavior in the context‐related fear conditioning test (Figure 1). These results suggest that co‐housed control mice have impaired learning and memory, similar to that of AD mice.

**FIGURE 1:**
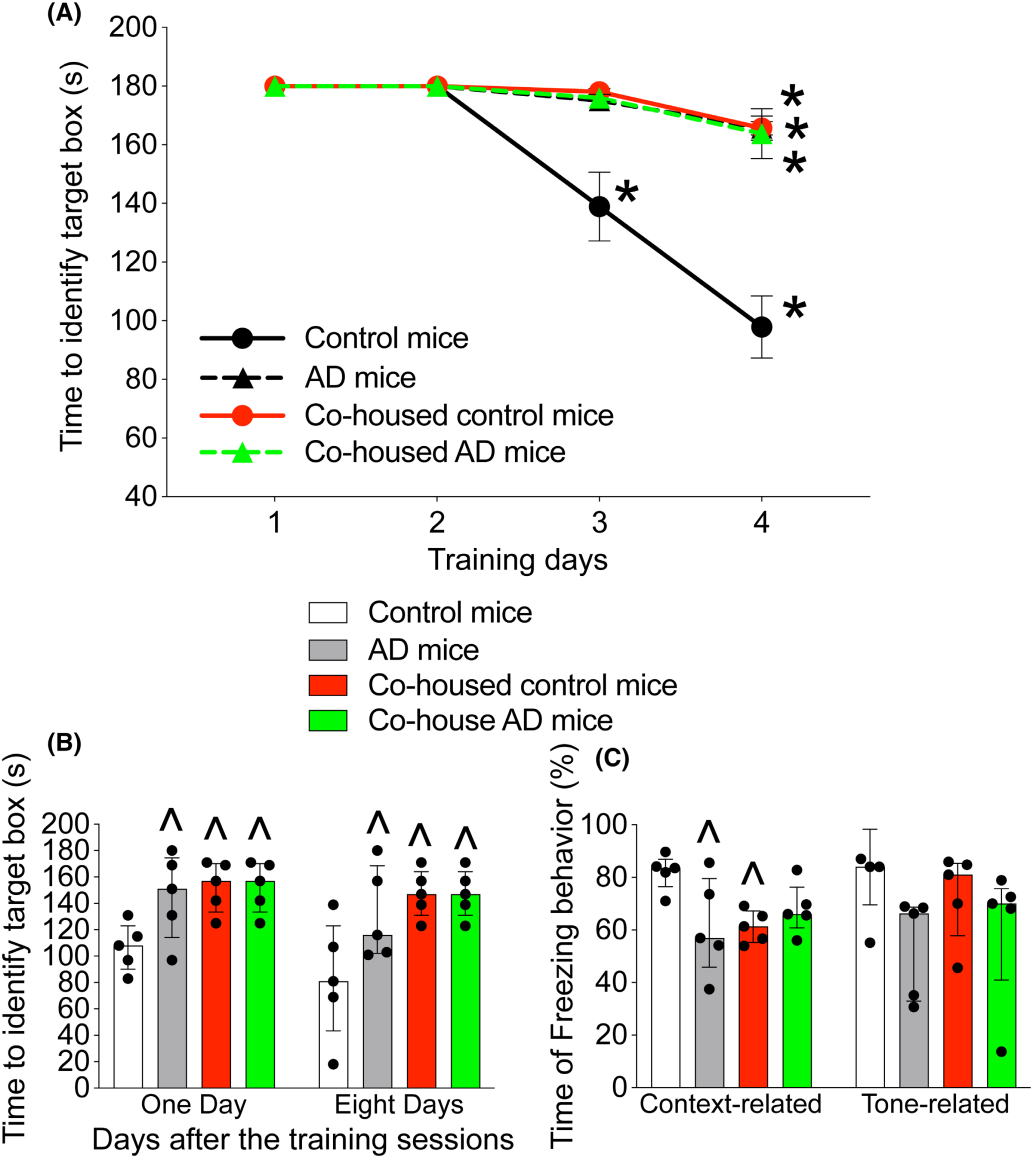
Cage‐sharing with mice with AD genes impairs learning and memory. (A) Training sessions of Barnes maze test. (B) 1 or 8 days after the training sessions in Barnes maze test. (C) Fear conditioning. Results are means ± SEM. (*n* = 5, panel A) or median ± interquartile range (*n* = 5, panels B and C). Data of each individual animal are also presented in the bar graphs. *p < 0.05 compared with the corresponding data on day 1 of the same group of mice. ^*p* < 0.05 compared with control group.

There was a difference in the taxonomic composition among the four groups of mice as indicated by β diversity parameters (Figure 2A,B). The composition at genus level was similar between 3xTg‐AD mice and co‐housed control mice (Figure 2C). For example, the relative abundance of g_anaeroplasma and g_clostridiales_family_XIV._incertae_sedis_unclassified in the co‐housed control mice was similar to that in the 3xTg‐AD mice but was higher than that in the control mice (Figure 2D,E).

**FIGURE 2:**
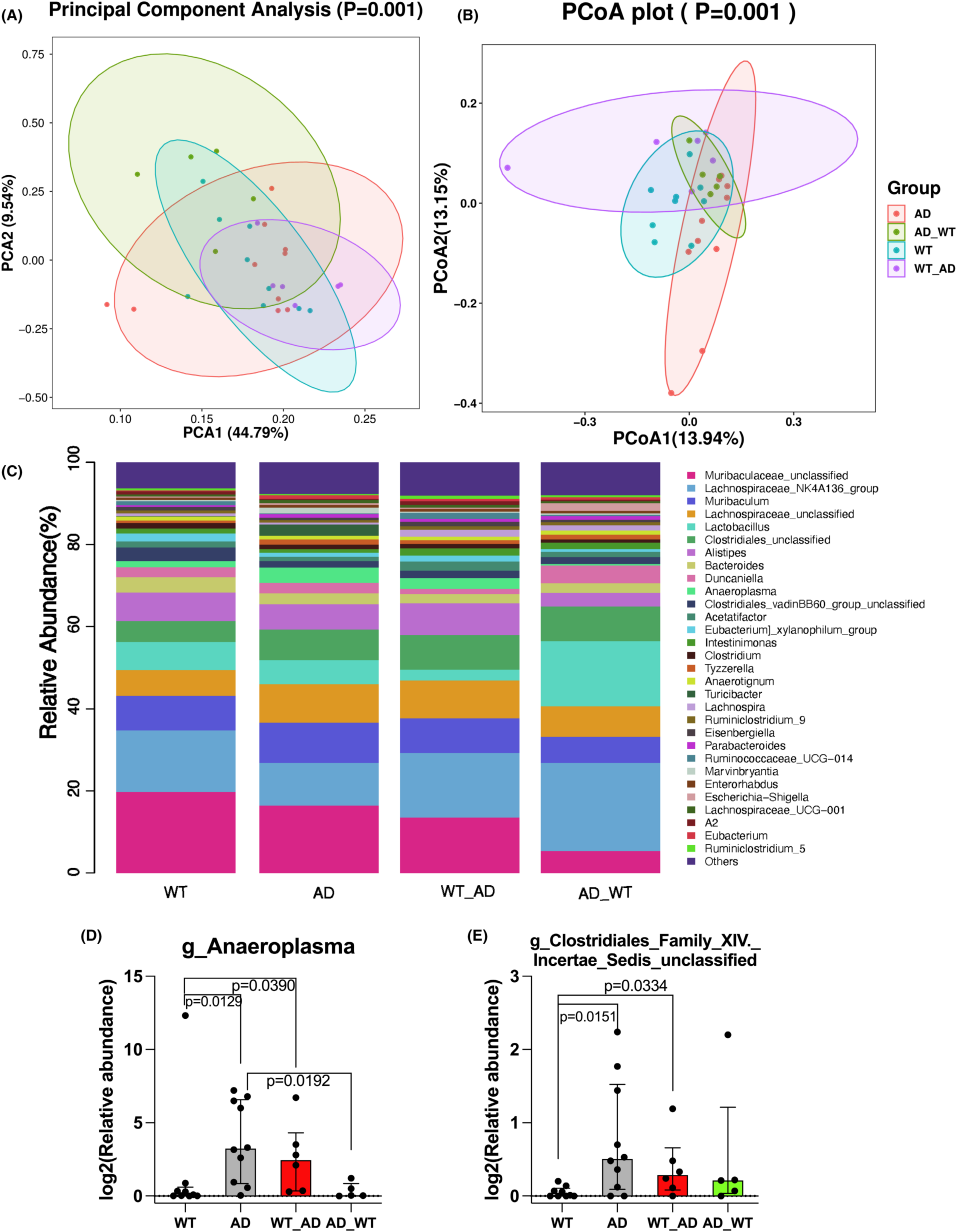
Comparisons of gut microbiota among the groups. (A and B) β diversity plots. (C) Relative abundance at genus level. (D and E) Comparison of two abundant representative genera among the groups. Results in panels D and E are median ± interquartile range (*n* = 5–10) with the presentation of data of each individual animal. AD, AD mice; AD_WT, AD mice co‐housed with control mice; WT, control mice; WT_AD, control mice co‐housed with AD mice.

The concentration of FITC‐dextran in the blood of 3xTg‐AD mice was higher than that of control mice. Similarly, co‐housed control mice had a higher FITC‐dextran concentration in the blood than control mice (Figure 3). These results suggest that 3xTg‐AD mice and co‐housed control mice have an increased intestinal permeability.

**FIGURE 3:**
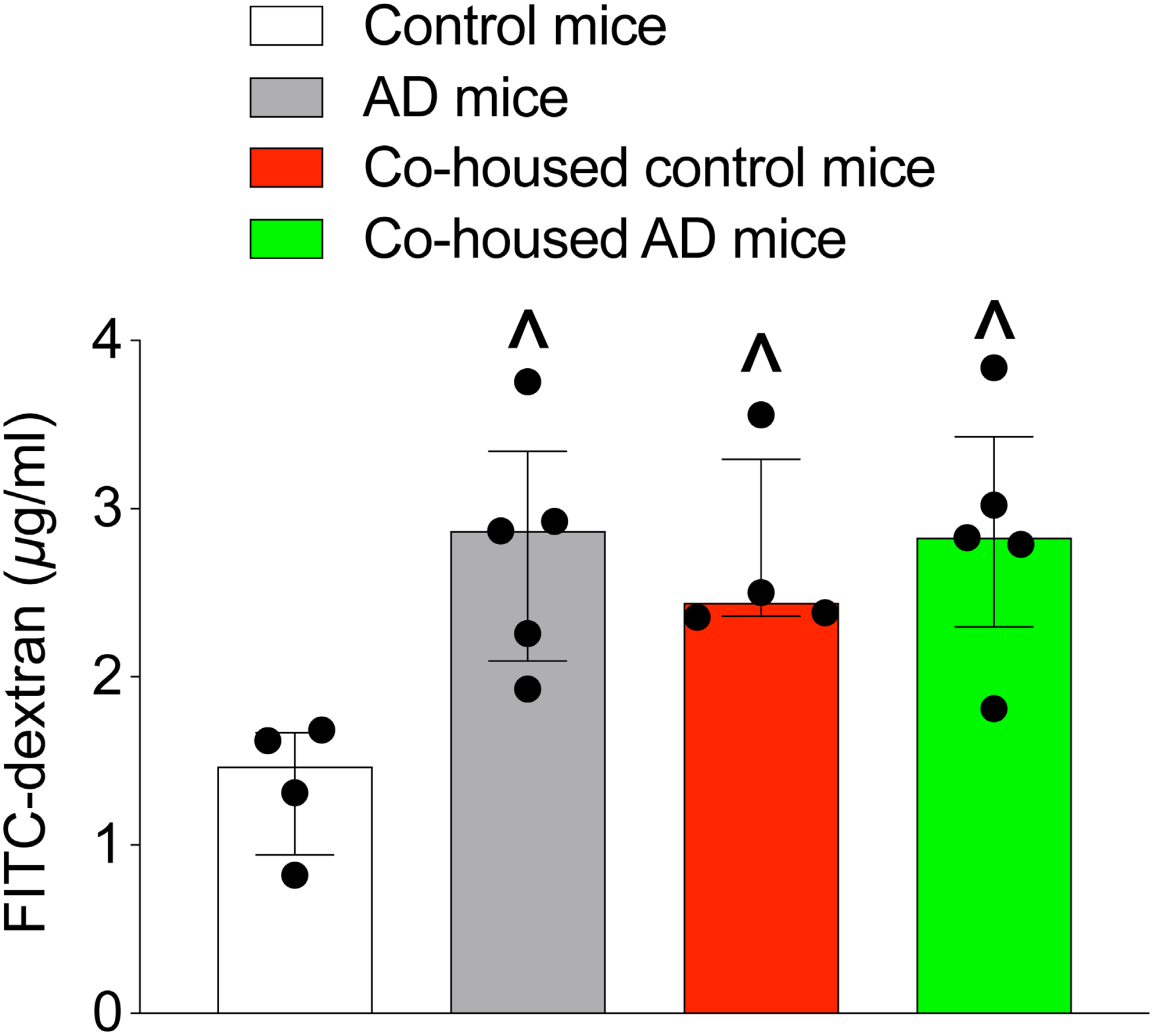
Gut permeability is measured by FITC‐dextran absorption into the blood. Results are median ± interquartile range (*n* = 4–5) with the presentation of data of each individual animal. ^*p* < 0.05 compared with control group.

Our results clearly indicate that co‐housed control mice develop learning and memory impairment. This provocative and novel finding suggests a transmission of this learning and memory impairment from co‐housed 3xTg‐AD mice to co‐housed control mice, which may present a good animal model for studying why spousal caregivers of patients with dementia have an increased risk for dementia later in life^10^ and why caring for a spouse with dementia worsens cognitive decline with aging.^15^

Stress along with shared lifestyle and space are proposed to contribute to the increased cognitive decline in the spouses caring for patients with dementia.^10^, ^11^ In our study, stress may not be a major factor for the impaired learning and memory in co‐housed control mice. 3xTg‐AD mice did not have surgery or pain that could have increased the stress or anxiety of the cage‐mate control mice.^16^, ^17^ In addition, co‐housed control mice did not need to provide care to their cage‐mate AD mice. As such, one possible route for the transmission of learning and memory impairment to co‐housed control mice is via gut microbiota because they share living space, which allows cross‐contamination via their feces.

Studies have shown that patients with AD have gut microbiota that is different from that of control participants.^5^, ^6^, ^7^ Interestingly, patients with mild cognitive impairment (MCI) have altered gut microbiota.^5^, ^6^, ^7^ These changes include a decrease in potentially protective microbiota, such as *Bacteroides*, and an increase in microbiota that can promote inflammation, such as *Prevotella*. There is a trend that the degree of gut dysbiosis is worsened with the disease stage from MCI to AD in our previous study.^7^ Similarly, many studies have shown altered gut microbiota in mice with AD‐like brain changes.^8^, ^9^ Interestingly, antibiotic or probiotic treatment of mice with genes for AD reduces Aβ accumulation in the brain and cytokines in the brain and blood.^18^, ^19^ The Aβ pathology was reduced in germ‐free AD transgenic mice.^20^ Fecal microbiota transplantation studies provide further evidence for the role of gut microbiota in AD‐like brain changes. The transplantation of healthy gut microbiota to AD transgenic mice reduces AD‐like brain changes.^9^ Transplanting fecal microbiota from patients with AD or mice with AD‐like brain changes to wild‐type mice increases endoplasmic reticulum stress in these wild‐type mice.^21^ Transplanting fecal microbiota from transgenic mice with AD genes also impairs learning, memory, and neurogenesis and induces neuroinflammation in wild‐type mice.^22^ Consistent with these findings, control mice living with 3xTg‐AD mice had a gut microbiota composition that was similar to that of 3xTg‐AD mice in this study.

Interestingly, 3xTg‐AD mice and control mice living with these AD mice had an increased intestinal permeability. Increased intestinal permeability has been shown in patients with dementia in a pilot study.^23^ Intestinal permeability measured also using FITC‐dextran is increased in endogenous melatonin reduction mice. These mice have neuroinflammation and increased total tau.^24^ Wild‐type mice receiving fecal transplant from mice with AD genes develop inflammation in their guts, suggesting a contribution of gut dysbiosis to the increased intestinal permeability.^22^ Obviously, increased intestinal permeability will allow chemicals including harmful chemicals from the gut to have access to the blood, which may be a mechanism to induce detrimental effects on the organs and tissues including the brain.

The 3xTg‐AD female mice were about 14 to 15 month old when they were tested in Barnes maze. Their learning was very significantly affected as reflected by poor performance during the training sessions. This result is similar to that of 12‐month old 3xTg‐AD female mice tested in the Morris water maze^25^ that, similar to Barnes maze, also tests the spatial learning and memory of animals.

Our study has limitations. First, as an animal model, we have not determined the minimal length of cohousing needed to impair the learning and memory of control mice. In this study, we simulated the human condition where spouses live together with patients for a long time. However, knowing the minimal length of cohousing will be useful to reduce the time of future experiments on identifying the mechanisms for the detrimental effects on the brain. Second, our findings suggest a role of gut microbiota change and increased intestinal permeability for the impairment of learning and memory in the co‐housed control mice. However, we have not identified which bacteria may contribute to these detrimental effects. Finally, 3xTg‐AD mice and co‐housed control mice have an increased intestinal permeability. This increase may be due to the increased permeability of gut mucosa, mucosa‐blood barrier, or both. Our study has not determined which impaired barrier function contributes to the increased intestinal permeability. Future studies will determine these unknowns.

In summary, living with AD mice for a long time impairs learning and memory of control mice. This co‐housing also alters their gut microbiota into an AD mouse‐like pattern and increases intestinal permeability in the control mice, pathological changes that have been suggested to contribute to the AD presentation in humans and animals.

## FUNDING INFORMATION

This study was supported by the Robert M. Epstein Professorship endowment (to Zhiyi Zuo) and Department of Anesthesiology, University of Virginia, Charlottesville, VA, USA.

## AUTHOR CONTRIBUTIONS

Zhiyi Zuo conceived the project; Jun Li, Weiran Shan, and Zhiyi Zuo designed the studies; Jun Li and Weiran Shan performed the experiments; Jun Li and Weiran Shan did initial data analysis and drafted methods section; Zhiyi Zuo performed the final analysis of the data and wrote the manuscript.

## CONFLICT OF INTEREST STATEMENT

The authors declare no conflicts of interest.

## Data Availability

The data are available upon a reasonable request.
